# Supplementary figures and images for: Widespread Exaptation of L1 Transposons for Transcription Factor Binding in Breast Cancer
Source: Int J Mol Sci. 2021 May 25;22(11):5625. doi: 10.3390/ijms22115625 (PMC8199441; doi:10.3390/ijms22115625)

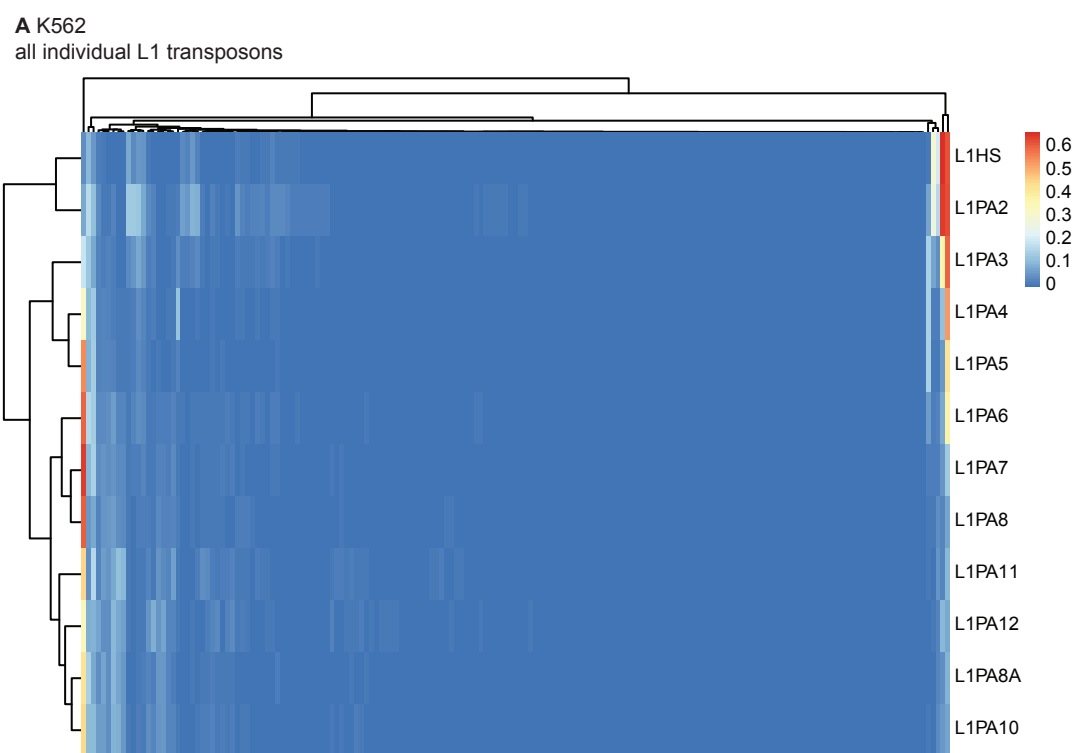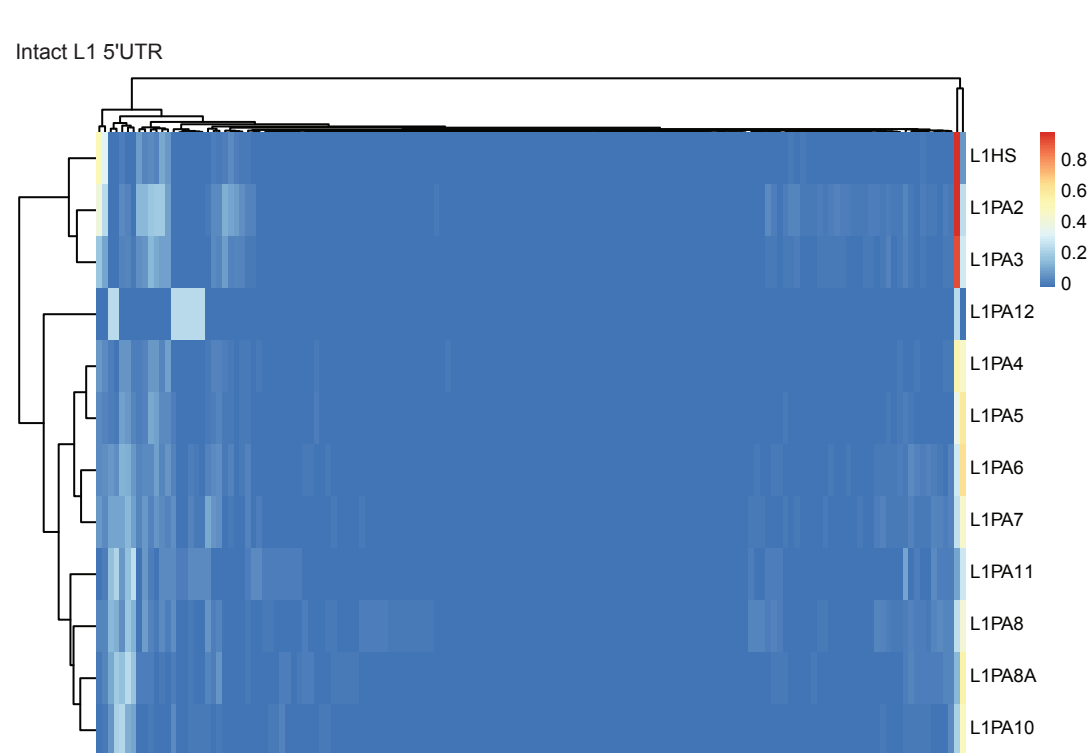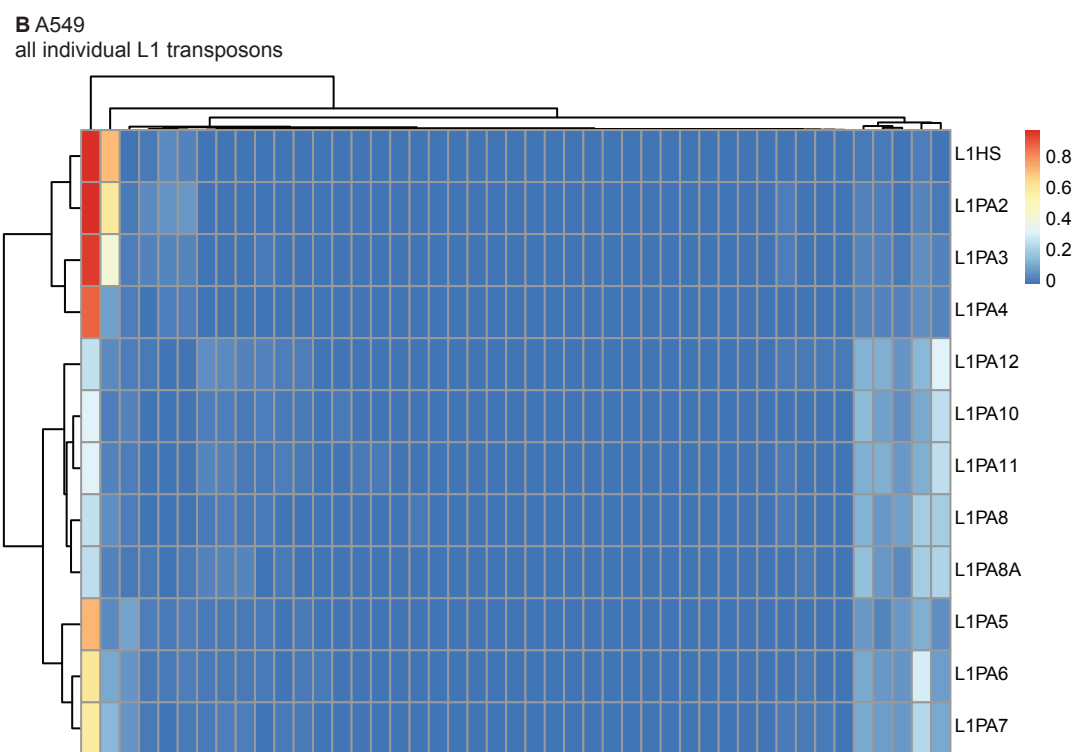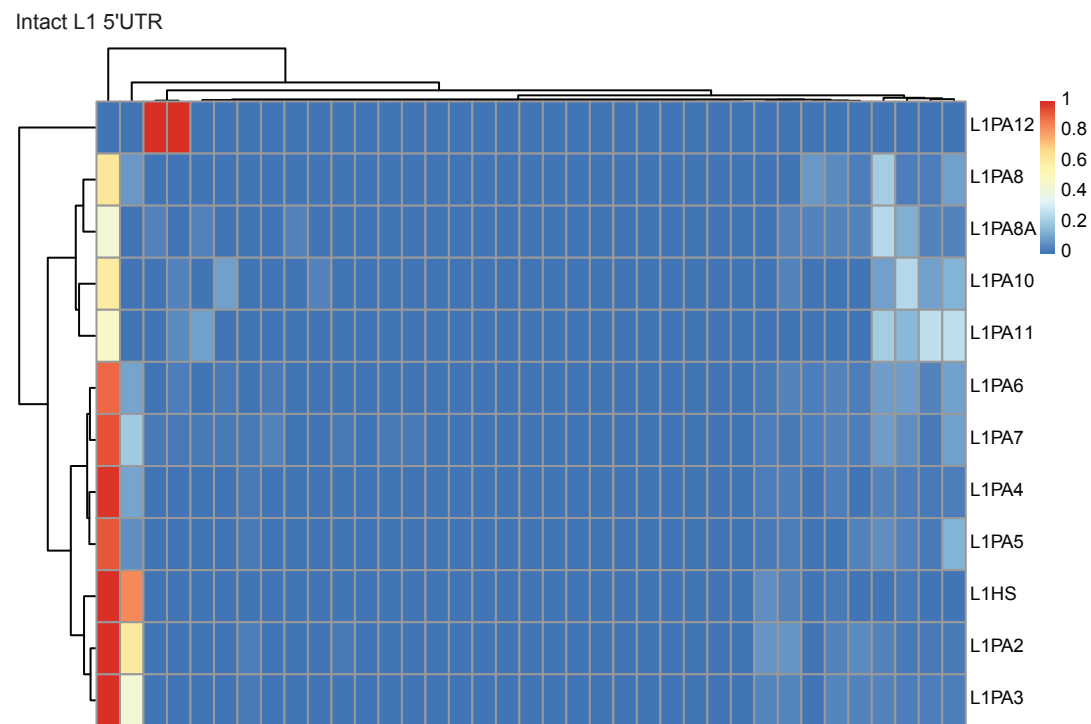

Supplement: Supplementary file 1 [file ijms-22-05625-s001.zip › Supp Fig S5.pdf]

A All individual L1 transposons

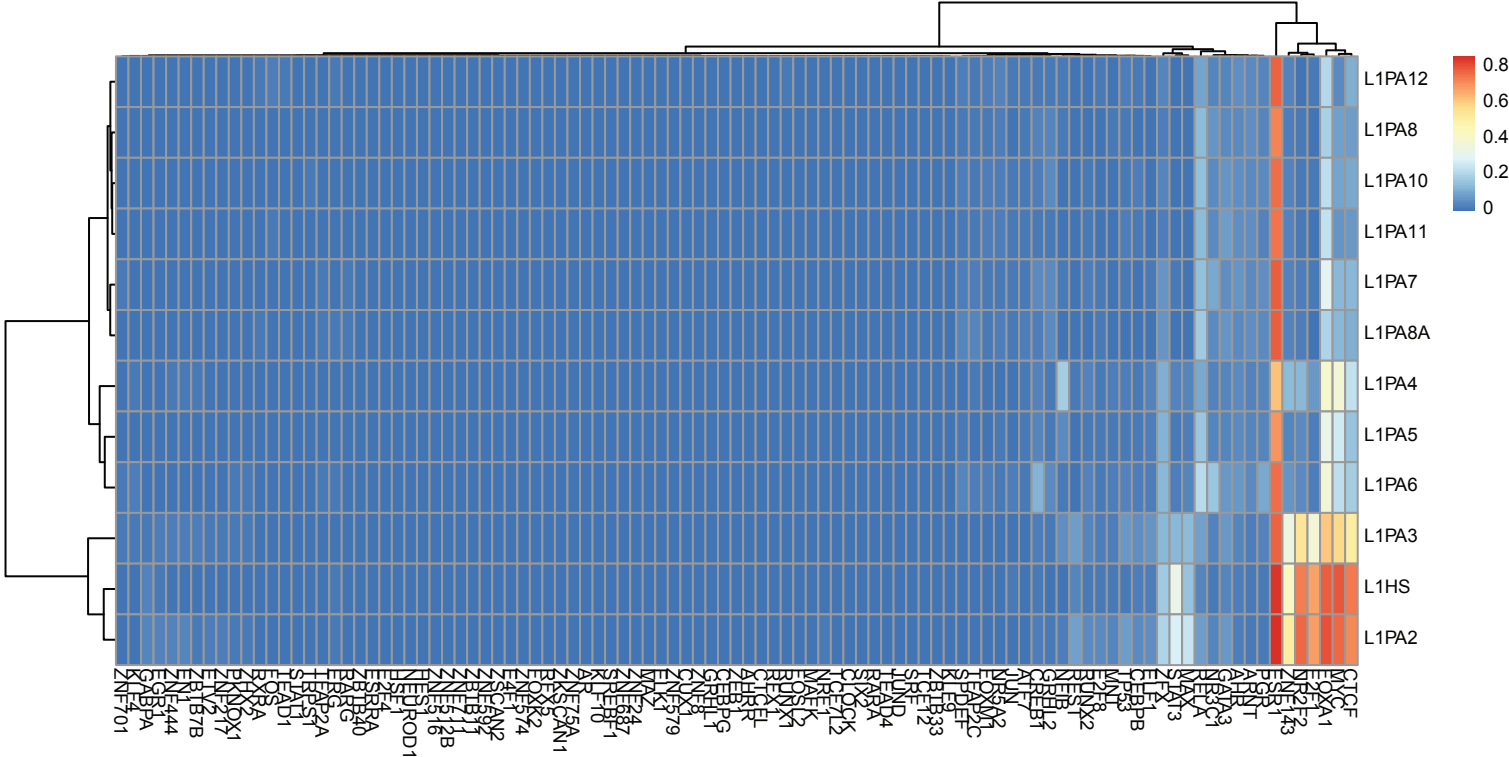

B Intact L1 5'UTR

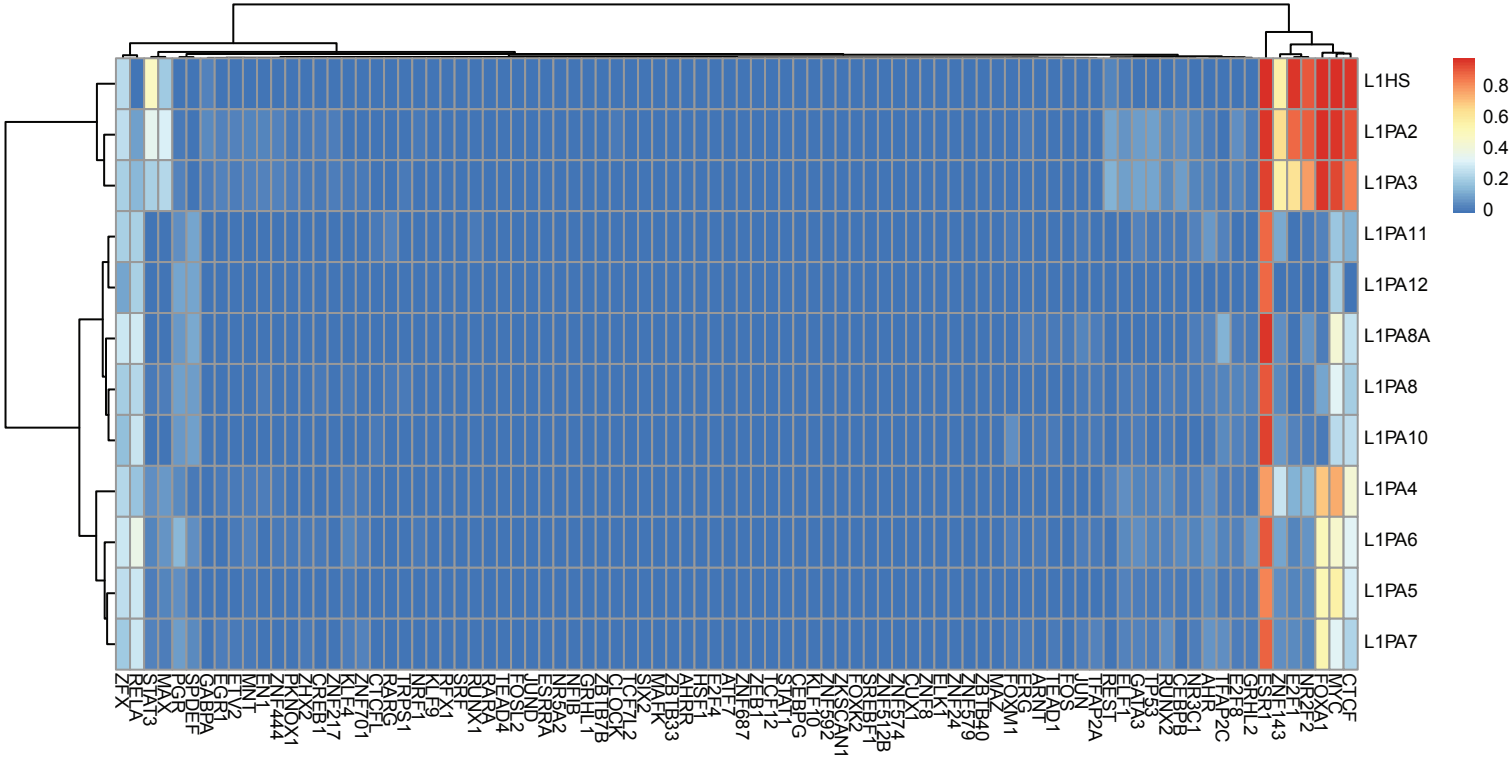

Supplement: Supplementary file 1 [file ijms-22-05625-s001.zip › Supp Fig S4.pdf]

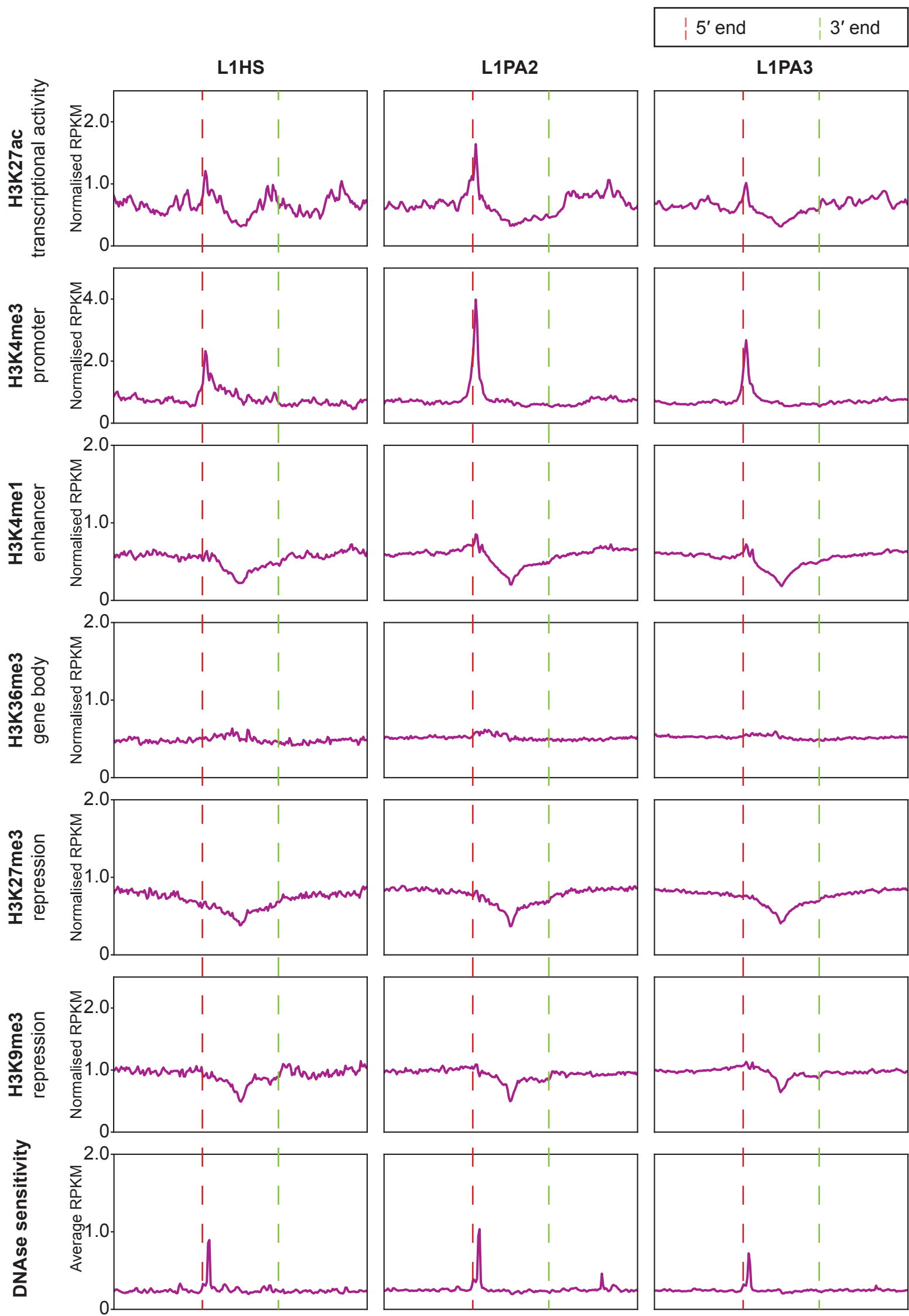

Supplement: Supplementary file 1 [file ijms-22-05625-s001.zip › Supp Fig S3.pdf]

**A** All individual L1 transposons

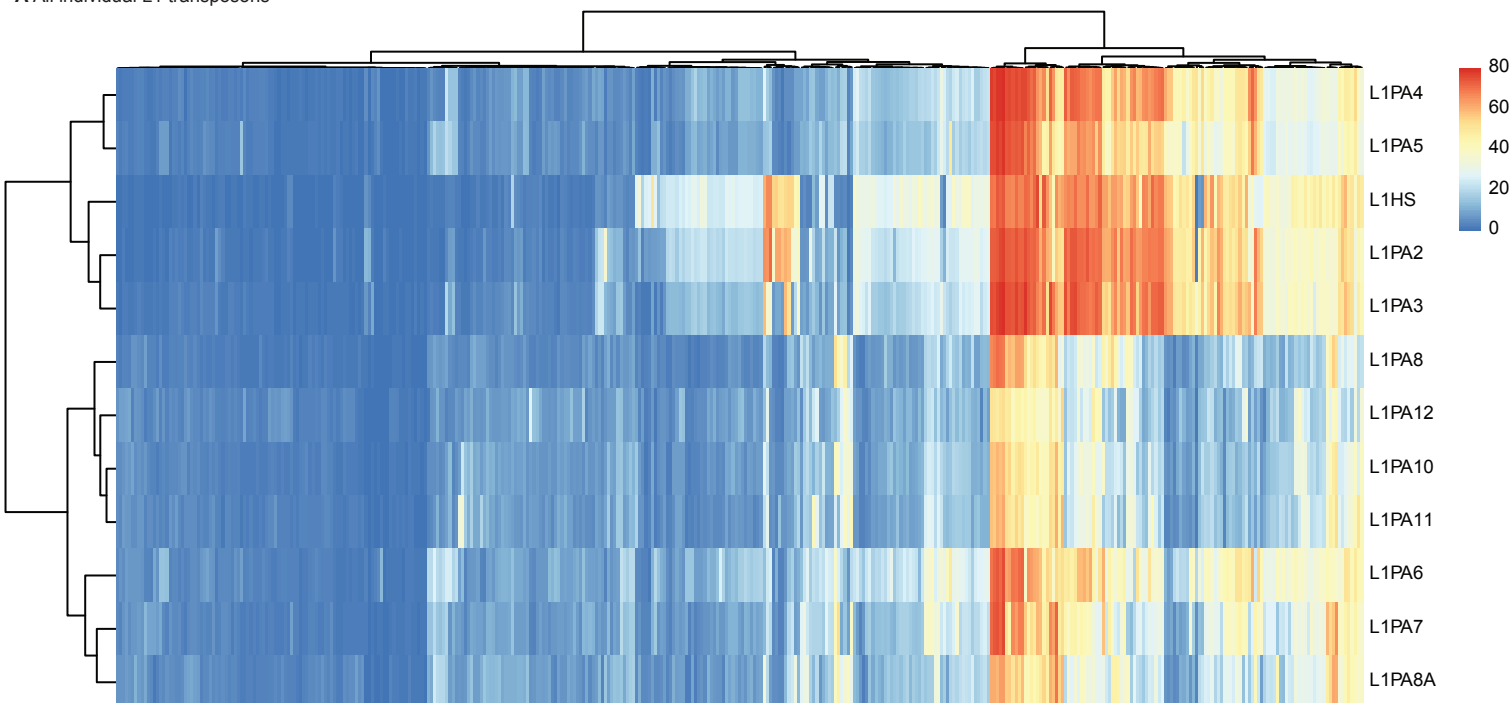

**B** Intact L1 5'UTR

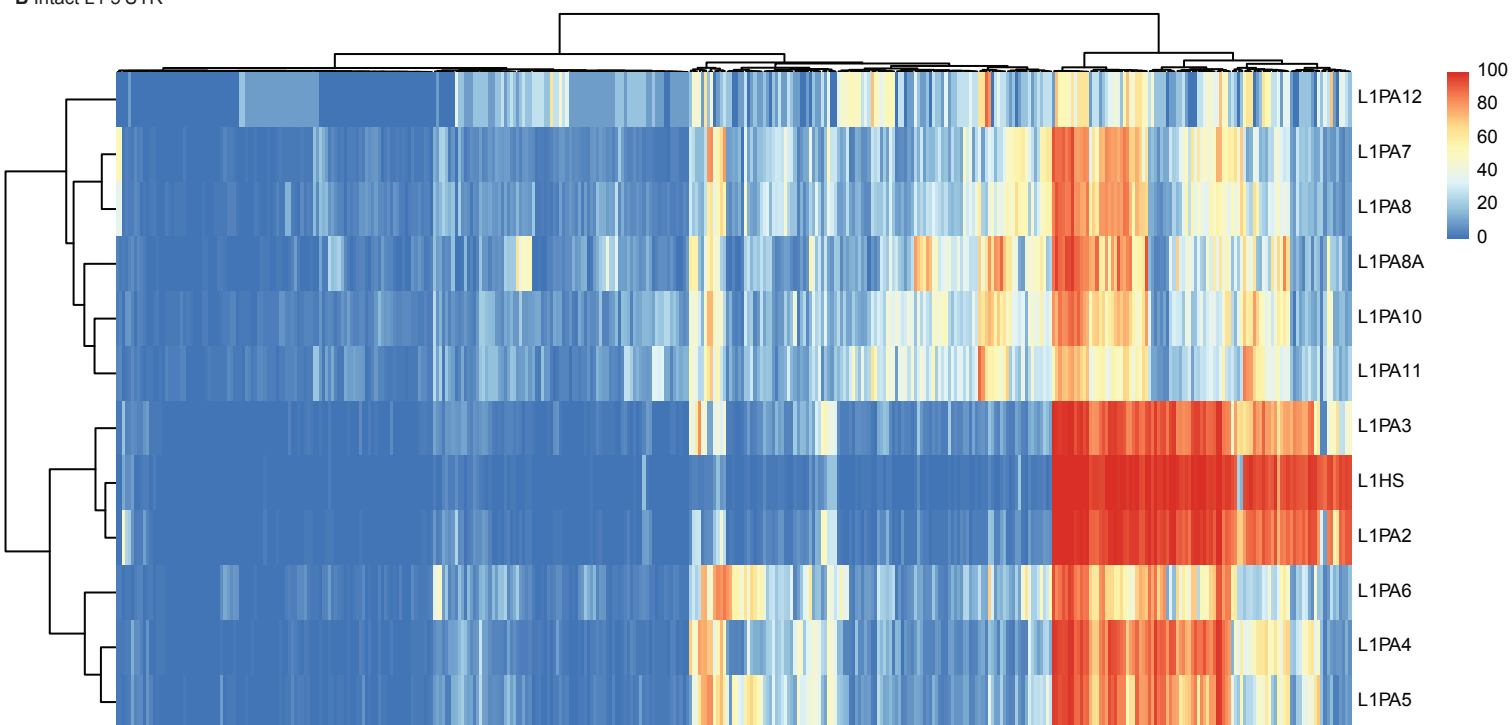

Supplement: Supplementary file 1 [file ijms-22-05625-s001.zip › Supp Fig S2.pdf]

Coverage

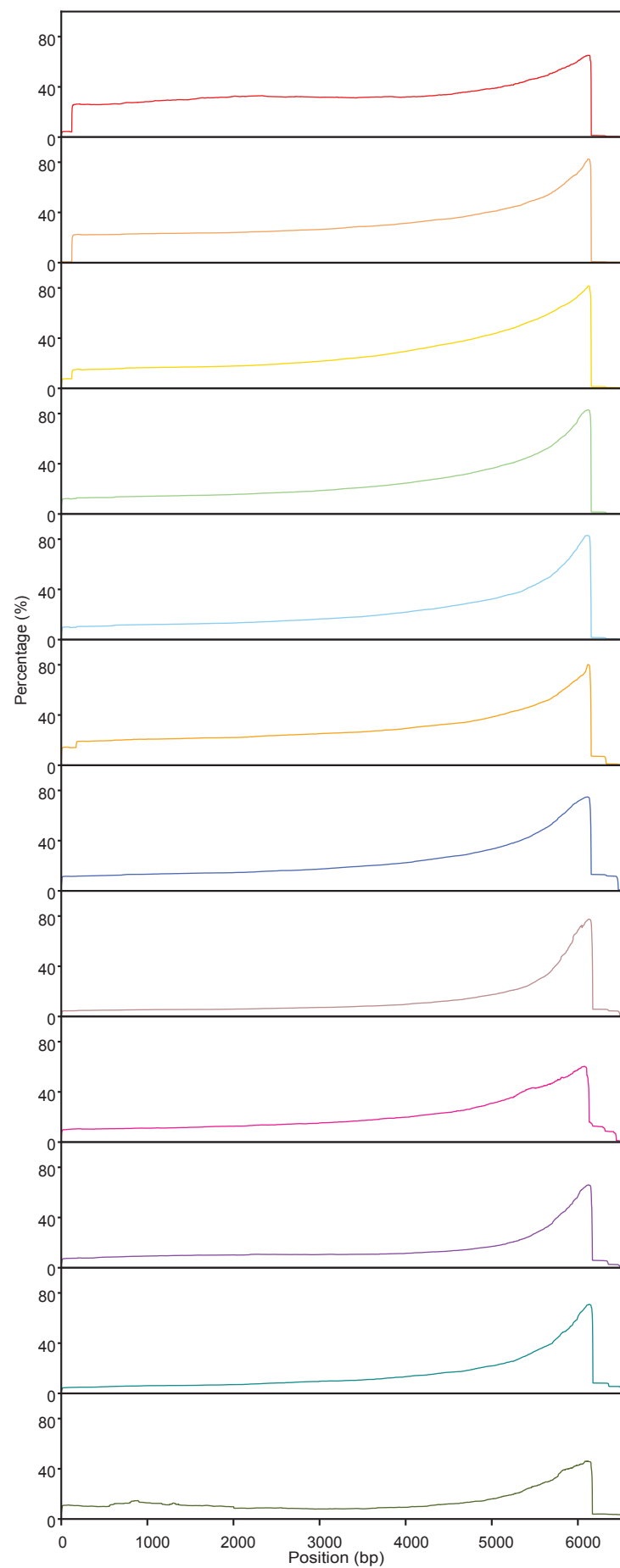

Length distribution

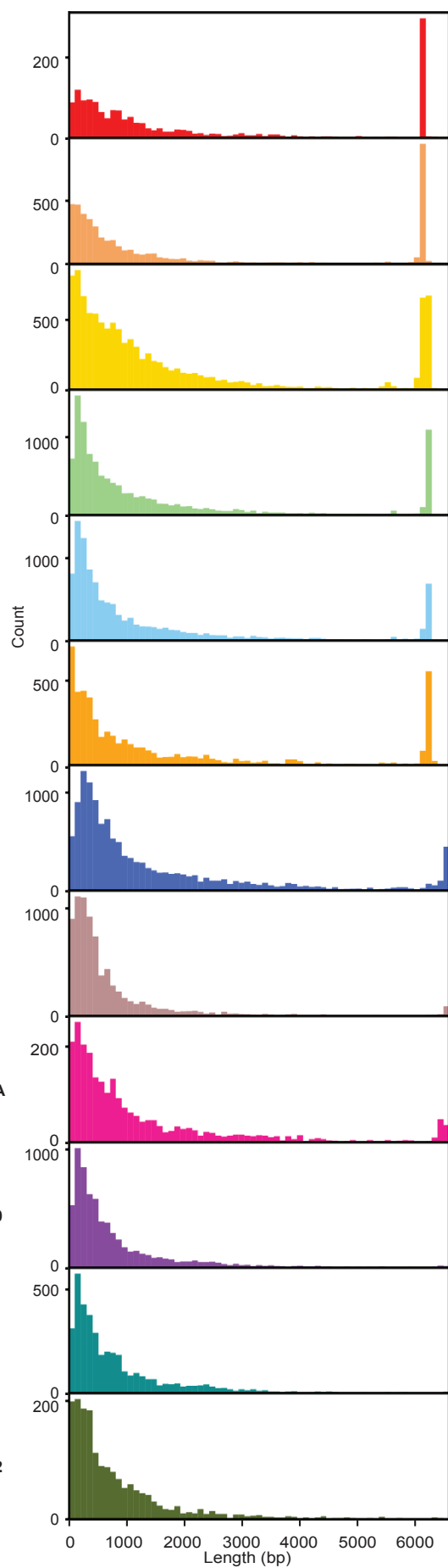

Supplement: Supplementary file 1 [file ijms-22-05625-s001.zip › Supp Fig S1.pdf]
